# Three-dimensional vascular and metabolic imaging using inverted autofluorescence

**DOI:** 10.1117/1.JBO.26.7.076002

**Published:** 2021-07-08

**Authors:** Shima Mehrvar, Soudeh Mostaghimi, Amadou K. S. Camara, Farnaz H. Foomani, Jayashree Narayanan, Brian Fish, Meetha Medhora, Mahsa Ranji

**Affiliations:** a University of Wisconsin–Milwaukee, Biophotonics Laboratory, Department of Electrical Engineering, Milwaukee, Wisconsin, United States; b Medical College of Wisconsin, Department of Physiology, Milwaukee, Wisconsin, United States; c Medical College of Wisconsin, Cardiovascular Research Center, Department of Anesthesiology, Milwaukee, Wisconsin, United States; d Medical College of Wisconsin, Cardiovascular Research Center, Department of Radiation Oncology, Milwaukee, Wisconsin, United States; e Florida Atlantic University, Department of Computer and Electrical Engineering and Computer Science, Boca Raton, Florida, United States

**Keywords:** vascular imaging, three-dimensional vessel network, label-free, fluorescence imaging, NADH, whole organ

## Abstract

**Significance:** Three-dimensional (3D) vascular and metabolic imaging (VMI) of whole organs in rodents provides critical and important (patho)physiological information in studying animal models of vascular network.

**Aim:** Autofluorescence metabolic imaging has been used to evaluate mitochondrial metabolites such as nicotinamide adenine dinucleotide (NADH) and flavine adenine dinucleotide (FAD). Leveraging these autofluorescence images of whole organs of rodents, we have developed a 3D vascular segmentation technique to delineate the anatomy of the vasculature as well as mitochondrial metabolic distribution.

**Approach:** By measuring fluorescence from naturally occurring mitochondrial metabolites combined with light-absorbing properties of hemoglobin, we detected the 3D structure of the vascular tree of rodent lungs, kidneys, hearts, and livers using VMI. For lung VMI, an exogenous fluorescent dye was injected into the trachea for inflation and to separate the airways, confirming no overlap between the segmented vessels and airways.

**Results:** The kidney vasculature from genetically engineered rats expressing endothelial-specific red fluorescent protein TdTomato confirmed a significant overlap with VMI. This approach abided by the “minimum work” hypothesis of the vascular network fitting to Murray’s law. Finally, the vascular segmentation approach confirmed the vascular regression in rats, induced by ionizing radiation.

**Conclusions:** Simultaneous vascular and metabolic information extracted from the VMI provides quantitative diagnostic markers without the confounding effects of vascular stains, fillers, or contrast agents.

## 1 Introduction

Damaged vasculature and the resulting impaired blood circulation in organs can cause pathological injuries, such as organ failure and stroke.^1^ Therefore, vascular imaging plays a pivotal role in diagnosis, follow-up of disease progression, and assessment of treatment efficacy.^2^ Assessment of vascular structure in rodent models is key to quantitate organ vasculature.^3^^,^^4^ This quantitation could be beneficial in analyzing pathological conditions, such as hypertension,^5^ diabetes,^6^ and retinopathy^7^ as well as changes induced by environmental or chemical agents such as radiation^8^ or drugs.^9^ Vascular imaging is also important to study therapeutic angiogenesis.^10^

The gold standard for vascular imaging of small animal organs is histology, which has a major limitation for obtaining a three-dimensional (3D) picture of structural components, e.g., the branching of a vascular tree.^11^ Additionally, using histology for vascular imaging of small animals requires the development of molecular tools such as specific antibodies^12^^,^^13^ or the development of transgenic mice expressing endothelial-specific markers.^14^ Imaging modalities such as micro-computed tomography (micro-CT),^15^ ultra-microscopy,^15^ near-infrared fluorescence imaging,^16^ magnetic resonance imaging,^17^ and ultrasound imaging^18^ are existing tools for vascular imaging in 3D, but they are complex and costly. Labeling with a contrast agent or filler is required for most of these vascular imaging technologies,^19^ each having its limitations. In some applications, a solvent must be used to optically clear the tissue and overcome the limiting 3D vascular image contrast, especially for high light-scattering organs like the kidney.^20^ Imaging systems typically provide information about just one biological marker that limits the capacity to decipher complex disease with multiple hallmarks such as cancer.^21^ For instance, positron emission tomography (PET) can be used to provide specific molecular information,^22^ while a hybrid imaging technology, such as PET-CT,^23^ acquires anatomical and molecular information but in turn comes with increased cost, acquisition time, and complexity.

We propose an approach that enables us to perform autofluorescence metabolic imaging that provides both metabolic and vascular information simultaneously. The presented method here is solely based on autofluorescence imaging emanating from the tissue. Fluorescence metabolic imaging techniques pioneered by Chance et al.^24^ have been developed to measure mitochondrial redox state [nicotinamide adenine dinucleotide (NADH)/flavine adenine dinucleotide (FAD)]. Fluorescence imaging or spectroscopy of metabolic indices provides 2D functional maps from the surface of tissues *in vivo* or *ex vivo*.^25^^–^^27^ 3D functional maps can be built using fluorescence cryo-imaging to provide a volumetric mitochondrial redox state of the tissue. However, to the best of our knowledge, optical metabolic imaging using autofluorescence has not been used to delineate the anatomy of the vasculature of organs.

In this study, we present a segmentation algorithm for detecting the vasculature, which is based on the autofluorescent properties of tissues. This novel technique enables vascular detection without the need for labeling vessels with contrast agents or stains. We termed the technique “vascular and metabolic imaging” (VMI). It relies on the foreground autofluorescence (NADH or FAD) that reveals the background vessel network devoid of such metabolic signatures. We hypothesized that the dark voxels are associated with the vasculature because the red blood cells quench the autofluorescence signals from NADH and FAD.^24^^,^^27^ We further postulated that our segmented vasculature from VMI can be used to quantify the 3D vascular network of whole organs, such as kidney, lung, heart, and liver. Remarkably, VMI, via autofluorescence, can produce both metabolic redox state and vascular information simultaneously that is currently unattainable with any other existing imaging tools. We validated our vascular detection approach by co-registering the VMI vessel images with the vessel images segmented from red fluorescence in a genetically modified rat kidney that preferentially expresses TdTomato in vascular endothelial cells. We also used a partial body irradiation (PBI) rat model with minimal bone marrow sparing to detect radiation-induced vascular regression in multiple organs as well as to demonstrate VMI utility as a biomedical research tool with potential clinical implications.

## 2 Methods and Materials

### 2.1 Animals and Sample Preparations

In this study, the vascular images were segmented based on autofluorescence images of rat organs. All the animal studies and experiments were approved by the Institutional Animal Care and Use Committee (IACUC) at the Medical College of Wisconsin. The studies were performed using two rodent species, rats, and mice. Lungs, the lateral lob of liver, and kidneys were harvested from non-irradiated and irradiated adult female WAG/RijCmcr rats and hearts from non-irradiated male C57BL/6J mice. For lung sample preparation, the airway of the lungs was first inflated by gravity with 1 to 2 mL fluorescein isothiocyanate–dextran (FITC-dextran, MW 150,000, 100  μM solved in water). In addition to the airway injection of the lungs, the sample preparation was similar for all organs. They were immersed in chilled liquid isopentane for a couple of minutes before transferring them to liquid N2. All samples were stored in a −80°C freezer until optical cryo-imaging was performed.

#### 2.1.1 Partial body irradiation in rats

This method has been developed to expose the total body of the rat, except for part of one hind leg, to ionizing irradiation delivered by x-rays. A minimal volume of bone marrow (∼8% of total marrow) is spared to repopulate the marrow compartment and allow the rat to survive the acute hematopoietic injury within the first 30 days after radiation. The delayed effects of radiation on the lung (radiation pneumonitis) manifest between 42 and 90 days after 10 Gy or higher doses, whereas the kidney damage (radiation nephropathy) is observed after 90 days. This sophisticated rat model is the first in rodents to express the acute and delayed syndromes of radiation exposure in the same animals.^28^^–^^30^ In brief, rats were placed in Plexiglas jig. One hind leg was carefully externalized and shielded with a lead block. A total dose of 7.5, 10, or 12.5 Gy x-rays (n=3/group) was delivered. Irradiation and dosimetry were conducted as described.^30^ Age-matched siblings (n=3) were not irradiated and served as non-irradiated controls. Rats were followed up to 101 days to record vascular changes in multiple organs. The animals were euthanized, and their kidneys, livers, and lungs were harvested. The lungs were inflated with an FITC solution introduced via the trachea before freezing. A high dosage of radiation is well-known to cause damaged endothelium and regression of vessel networks.^31^^–^^34^ Our well-established, radiation-induced animal injury model provides an ideal system to demonstrate the sensitivity and efficacy of the algorithm to detect vascular damage in multiple organs.^35^^,^^36^

#### 2.1.2 CDH5-cre recombinase rat

For validation purposes, a transgenic rat, expressing the fluorescent protein TdTomato in vascular endothelium, was used. CDH5-cre recombinase rats were performed at the Genome Rat Resource Center at the Medical College of Wisconsin under protocols approved by the IACUC. Briefly, a 2.5-kbp PCR fragment of the rat genomic DNA encompassing Cdh5 promoter was cloned upstream of the codon-optimized HA-tagged Cre (iCre) and this expression cassette was subcloned into a sleeping beauty (SB) transposon vector.^37^ The SB method of transpositional transgenesis was used to produce transgenic Sprague Dawley (Crl:SD, Charles River Laboratories) rats by pronuclear microinjection as we have previously described.^38^^,^^39^ Three transgenic founders were produced, one of which demonstrated robust endothelial-specific Cre expression when crossed to the TdTomato reporter knock-in rat (Horizon). Both the Cdh5-Cre and TdTomato reporter knock-in rat were backcrossed to the WAG/RijCmcr inbred strain for four generations and then intercrossed for the studies presented herein.

### 2.2 3D Fluorescence Metabolic Cryo-Imaging

The 3D fluorescence cryo-imager system was custom-designed in the Biophotonics Laboratory at the University of Wisconsin Milwaukee. The system captures 3D NADH and FAD fluorescent signals of frozen organs/tissues. The flash-frozen sample is stored in −80°C freezer to ensure the preservation of the metabolic state of the tissue. A complete description of the system can be found in our recent cryo-imaging studies.^26^^,^^40^ Briefly, a mercury arc lamp (200 W lamp, Oriel, Irvine, CA, in the light source from Ushio Inc., Japan) is used as the light source. Appropriate optical filters at selected wavelengths are utilized to excite the specific fluorophores from the surface of the frozen tissue. For the NADH channel, excitation and emission filters were set at 350±80  nm (UV Pass Blacklite, HD Dichroic, Los Angeles, CA) and 460±50  nm (Chroma, Bellows Falls, VT), respectively. The excitation and emission filters for the FAD channel were set at 437±20  nm (Omega Optical, Brattleboro, VT) and 537±50  nm (Omega Optical), respectively. Lungs were also imaged using FITC specific optical filter sets: excitation at 494±20  nm (Edmund Optics) and emission at 537±50  nm (Omega Optical) for airway detection. For imaging Td-Tomato kidney, besides the regular NADH channel, we also used a red channel of imaging, with the excitation and emission filters set at 545±25  nm (Chroma) and 645±50  nm (Chroma), respectively. All filters are controlled by two motorized filter wheels (Oriental Motor Vexta Step Motor PK268-01B). The emitted fluorescent signals are captured with the image recordings system (CCD camera, QImaging, Rolera EM-C2, 14 bit).

The 3D NADH and FAD cryo-images, representing mitochondrial redox state of tissues, were analyzed using a code written in MATLAB. Calibration was performed using a flat-field image of both NADH and FAD channels. The 3D-rendered redox ratio (RR) image (NADH/FAD) was calculated voxel-by-voxel. Also attempts to understand the heterogeneity of the tissue were made to correlate the RR with the anatomy of the hearts^41^ and kidneys.^40^ The following section describes how the autofluorescence images provide the structural features of the vascular network of the organs.

### 2.3 Vascular Segmentation from Autofluorescence Images

**Fig. 1:**
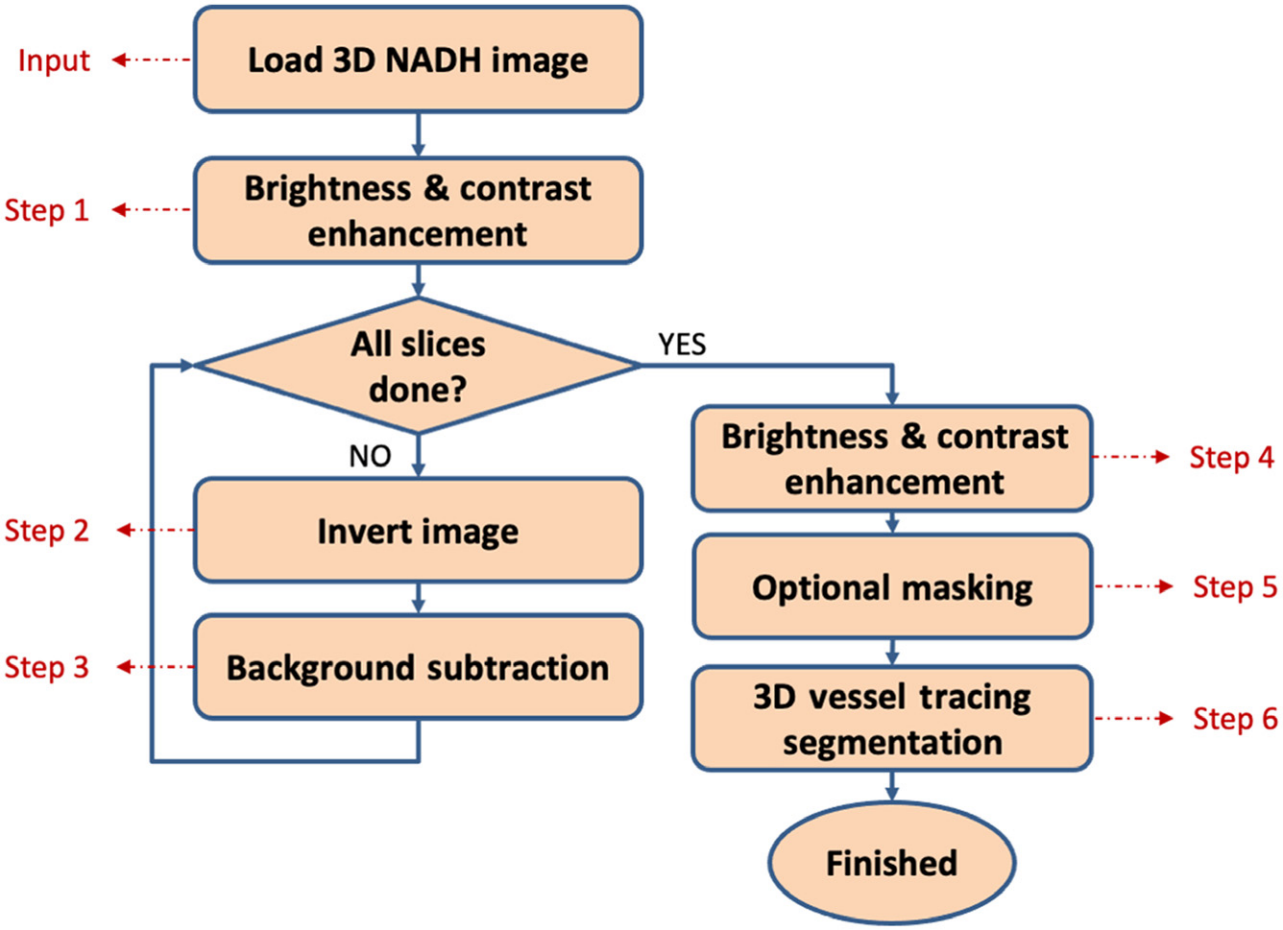
Algorithm flowchart for background vasculature segmentation from fluorescence images. After loading the 3D stack of images, the brightness and contrast are adjusted to have an enhanced image. Then for each slice, the image is inverted, and the background is subtracted. Additional contrast enhancement and optional masking of the unwanted regions based on the tissue type is done on the 3D image. The reconstructed 3D vasculature can be fed to 3D vessel tracing algorithms for quantification purposes.

Figure 1 shows the flowchart of the proposed algorithm that we used to segment the background vasculature from the foreground 3D autofluorescence images. A simple implementation steps in FIJI^42^ can be found in Table S1 in the Supplementary Material. The standard preprocessing normalization steps in fluorescence cryo-imaging, such as flat-field calibration is not needed before vascular segmentation because the intensity adjustment in step 1 normalizes between-sample variations, and the background subtraction in step 3 will remove the uneven illumination.

Below is the detailed sequence of steps carried out to obtain and reconstruct a vascular network from the inverted fluorescent image.

**Fig. 2:**
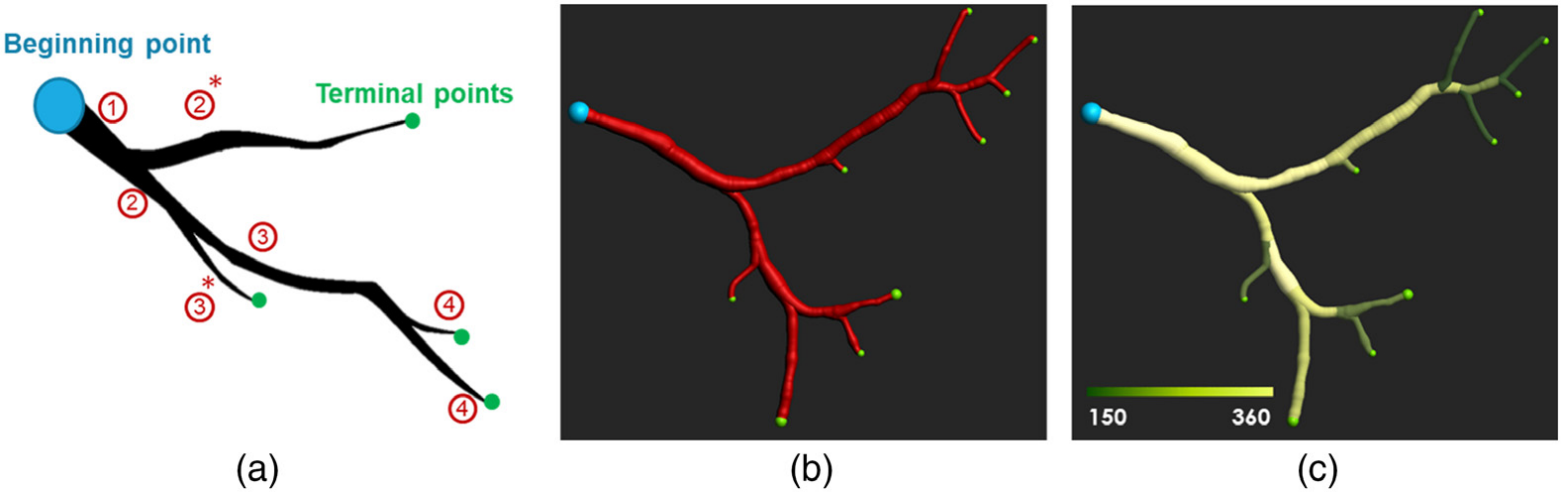
Quantification of vascular markers. (a) A schematic view of a branch: a vessel tracing algorithm traces the path from the beginning point to the terminal points. The branch depths are increasing in each bifurcation. (b) A simple branch illustrated with its terminal points and (c) the branch color-coded with vessel diameter (μm).

Step 1. *Brightness and contrast adjustments.* The brightness and contrast of images are adjusted by remapping intensity values to the full range of 16-bit images, i.e., adjusting the minimum voxel intensity of the image to zero and maximum intensity to $2^{16}=65,536$. The intensity of 3D fluorescence images is adjusted to the whole volume intensity range, and this step is performed on 3D images. The captured autofluorescence intensity might be different from samples to samples. This step of the algorithm is designed to specifically normalize the variations in the intensity of images from various samples by adjusting the intensity of images to similar intensity range. This will also sharpen the differences between the black and white voxels, i.e., enhancing the image contrast. Contrast enhancement is generally used to make objects in an image more distinguishable.
Step 2. *Image inversion.* In our application, vascular network elements are dark voxels. The inverted image (negative contrast) displays the vasculature as bright voxels. This step is performed on each 2D slice separately.
Step 3. *Background subtraction.* A background subtraction algorithm known as rolling-ball background correction^43^ is used in the next step. The rolling ball radius in each organ should be at least set to the largest vessel radius that we expect the organ possesses. The radius of larger vessels can be estimated by manual measurements in the 2D image containing the larger vessel. Background subtraction is traditionally used in fluorescence microscopy to isolate bright objects from an uneven illumination.^44^ This step is performed on each 2D slice separately.
Step 4. *Brightness and contrast adjustments*. Final contrast enhancement is also done on the 3D structures by repeating step 1, i.e., intensity adjustment.
Step 5. *Optional masking*. Before feeding the 3D vasculature images to the tracing algorithm, based on the organ, some masking is also required. The heart cavities (atria and ventricles) were masked out using a thresholding mask calculated from the original NADH images. Also, for the kidneys, the segmented vasculature from the medullary region was masked to ensure total removal of false segmentation originating from renal tubules within the medullary region. This removal of the medullary voxels removed a negligible portion of the segmented vascular network (see Fig. S1 in the Supplementary Material), while making sure that the segmented vasculature did not contain renal tubules.
Step 6. *3D vessel tracing algorithm.* By tracing the 3D vessel networks, we can track, measure, and quantify the vasculature. We used Imaris 9.5 software (Bitplane Inc.) and their filament tracing algorithm, which is based on local intensity contrast. It traces and finds the path from the largest starting point(s) to the smallest terminal points [Fig. 2(a)]. Then the vessel branches can be quantified to provide various vascular biomarkers. For instance, the diameter of a vascular branch [Fig. 2(b)] can be calculated as illustrated in Fig. 2(c).

If the structure comes from high-intensity voxels such as airway in FITC airway injected lungs and red fluorescence images in TdTomato rat kidneys, the same segmentation algorithm without step 2 (image inversion) can be applied.

### 2.4 Validating VMI Using TdTomato Rats

A genetically modified rat model expressing TdTomato primarily in vascular endothelial cells was utilized to image the vasculature in kidneys. Histological assessment of rat kidneys was also done to visualize TdTomato expression in endothelial cells of these rats using an antibody for TdTomato. The capability of the 3D fluorescence cryo-imaging to acquire images from multiple channels simultaneously allows us to have both red and NADH fluorescence of the kidney. We used the foreground vasculature extracted from red fluorescence as proof for examining the vasculature segmented from the NADH channel using VMI. The co-registration of the vascular network extracted from the two channels validates the proposed method of our vascular segmentation.

One of the common metrics for evaluating the quality of image segmentation is the Dice coefficient, which measures the overlap/merge between the ground truth and the test.^45^ For calculating the Dice coefficient, we let the 3D volume be represented by the point set $X=\{x_{1},\ldots ,x_{N}\}$, where N is the total number of voxels. We let the red vasculature be represented by the partition $V_{\text{red}}$ of X with assignment function $f_{\text{red}}(x)$, i.e., voxel intensity at x, and we let the VMI vasculature be represented by the partition $V_{\mathrm{vmi}}$ of X with assignment function $f_{\mathrm{vmi}}(x)$. Then the Dice coefficient is defined by

$$\text{Dice}=\frac{2|V_{\text{red}}\cap V_{\mathrm{vmi}}|}{\left|V_{\text{red}}|+|V_{\mathrm{vmi}}\right|}=\frac{2\sum_{i=1}^{N}f_{\text{red}}(x_{i})f_{\mathrm{vmi}}(x_{i})}{\sum_{i=1}^{N}f_{\text{red}}^{2}(x_{i})+\sum_{i=1}^{N}f_{\mathrm{vmi}}^{2}(x_{i})},$$ (1)

where the numerator represents the common elements between the two images. To quantify $\left|V_{\text{red}}\right|$ and $\left|V_{\mathrm{vmi}}\right|$, we use the squared sum operation. There is a multiplication by a factor of 2 in the numerator because the denominator counts the common elements twice.

The branching structure of the VMI vasculature can also be compared with red fluorescence vasculature. Murray^46^ proposed an optimization theory that the fundamental structure of a vascular tree should be such that it minimizes work. Murray’s law states that a branch that follows the “minimum work” hypothesis should also follow the equation:

$${\left(D_{p}\right)}^{3}=\underset{d}{\sum }{\left(D_{d}\right)}^{3},$$ (2)

where $D_{p}$ indicates the diameter of a parent vessel, and $D_{d}$ indicates the diameter of the d’th daughter vessel coming from the parent p. Equation (2) means that the cubed diameter of a parent vessel is equivalent to the sum of the cubed diameter of its daughter vessels.

After employing the tracing algorithm using filament tracing in Imaris software, we used the information on the depth of the vessels to define the parents and daughters. The depth of a vessel increases every time a bifurcation happens in the branch. Therefore, all vessels with depth k+1 are the daughter vessels of the parent vessels with depth k and Murray’s law can be written as

$$\underset{p}{\sum }{\left(D_{p_{k}}\right)}^{3}=\;\underset{d}{\sum }{\left(D_{d_{k+1}}\right)}^{3},$$ (3)

where $D_{p_{k}}$ indicates the diameter of the p’th parent vessel at depth k, and $D_{d_{k+1}}$ indicates the diameter of the d’th daughter vessel at depth k+1. Now, we can look at the relationship between the parent vessel diameters with their daughters’ diameter by having the depth information of the vessels. The summation of the cubed diameter of all the vessels at each depth [parents on the left side of Eq. (3)] is then compared with the summation of the cubed diameter of all the vessels at the next depth [daughters on the right side of Eq. (3)]. The vasculature follows Murray’s law if this relationship is significantly linear and has a linear fit close to the identity line.

Notably, using the depth to find the parent–daughter relationship in vessels can impose an unavoidable error by making the left side of Eq. (3) higher than the real value. The reason is that the terminal branches from lower depths [asterisks in Fig. 2(a)] are considered as parent vessel while there are no corresponding daughter vessels in the next depth.

## 3 Results

### 3.1 3D Vascular and Metabolic Imaging Using Autofluorescence

**Fig. 3:**
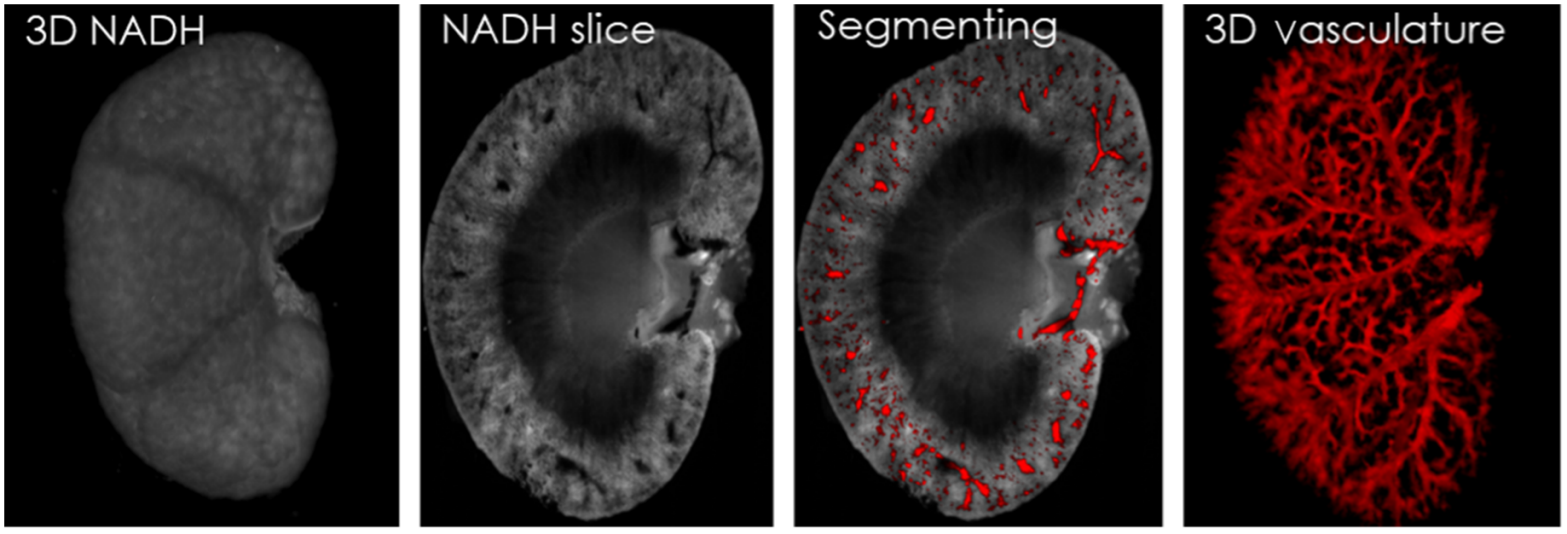
A background vasculature is segmented from a foreground autofluorescence image of a kidney. For a rat kidney, a 3D raw image of NADH fluorescence is shown. A sagittal slice view of raw kidney image is chosen, and the segmented vasculature from dark voxels is shown in red and merged with the raw slice to show the localization of the vascular pixels in the image. The 3D vasculature is reconstructed from all 2D segmented pixels. The 3D rendered images of NADH and vasculature can be found in Video S1 (Video S1, 685 kB, MP4 [URL: https://doi.org/10.1117/1.JBO.26.7.076002.1]) and Video S2 (Video S2, 471 kB, MP4 [URL: https://doi.org/10.1117/1.JBO.26.7.076002.2]), respectively.

Figure 3 supports our hypothesis that a foreground fluorescence image can be inverted to reveal the vasculature of an organ like the kidney. The 3D raw NADH (excitation at 350 nm and emission at 460 nm) image of a kidney, a sagittal slice of the kidney, and the segmented vasculature on one slice are illustrated in Fig. 3. The stack of 2D vascular images was reconstructed to generate the 3D vascular images of the whole kidney.

**Fig. 4:**
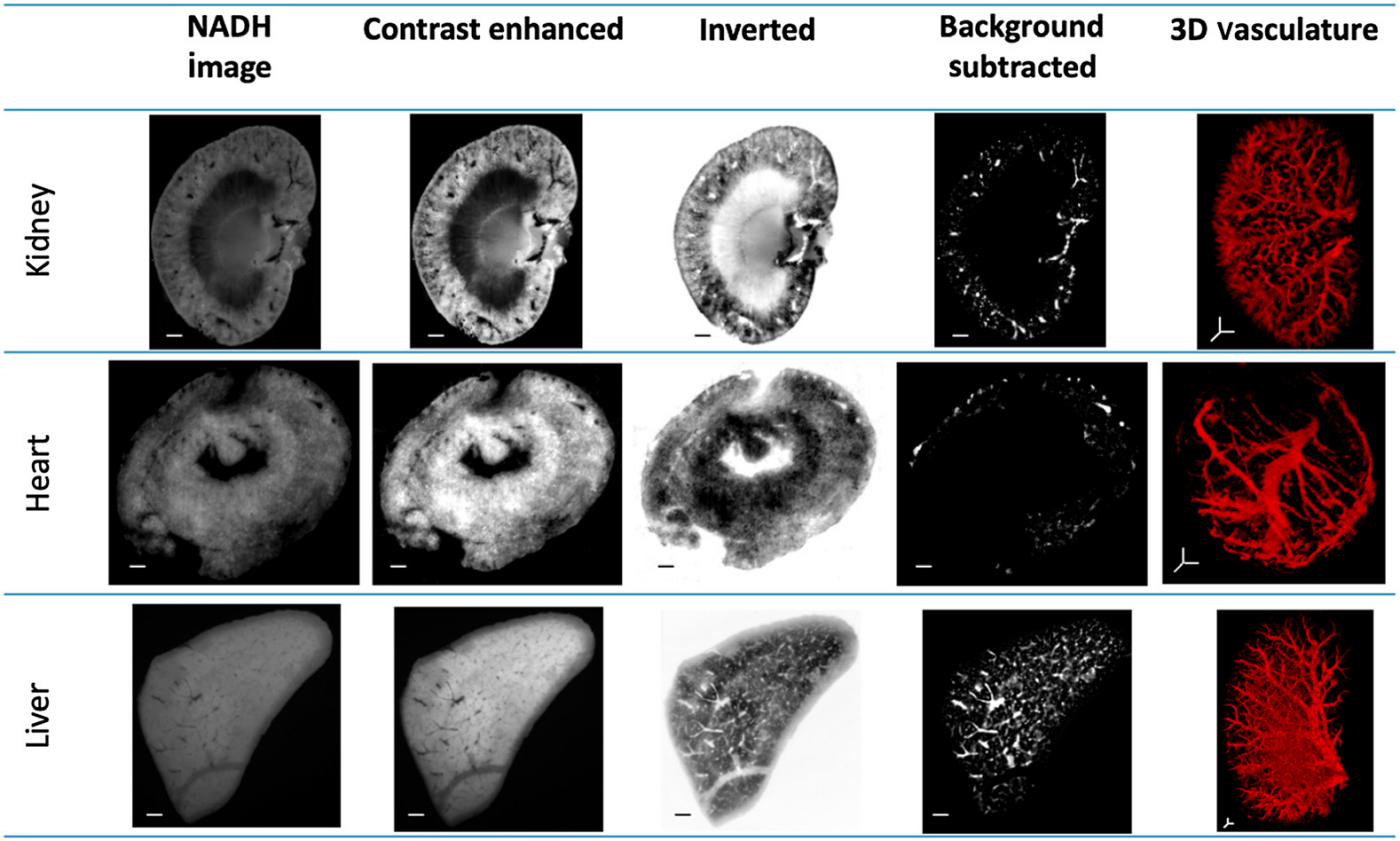
VMI can be used to segment vascular networks of multiple organs, including kidney, heart, and liver. An example slice in each step of the algorithm is illustrated for each organ, following which the 3D vasculature is reconstructed. The scale bars for kidney, heart, and liver are 600, 400, and 1000  μm, respectively. Videos from the 3D rendered vasculature images can be found in Videos S2 for kidney (Video S2, 471 kB, MP4), Video S3 for heart (Video S3, 470 kB, MP4 [URL: https://doi.org/10.1117/1.JBO.26.7.076002.3]), and Video S4 for liver (Video S4, 482 kB, MP4 [URL: https://doi.org/10.1117/1.JBO.26.7.076002.4]).

VMI was also applied to other organs, such as heart and liver. Figure 4 shows selected representative slices for the step-by-step images of the algorithm for each organ: kidney, heart, and liver. In step 1, the contrast and brightness of the images are enhanced. The inverted images of one slice of each organ can be seen in step 2. Note that now, the feature of interest (vasculature) is bright in the image. A background-subtracted image of the slice can be seen in step 3. The resulting 3D vascular images are reconstructed from the stack of 2D images.

**Fig. 5:**
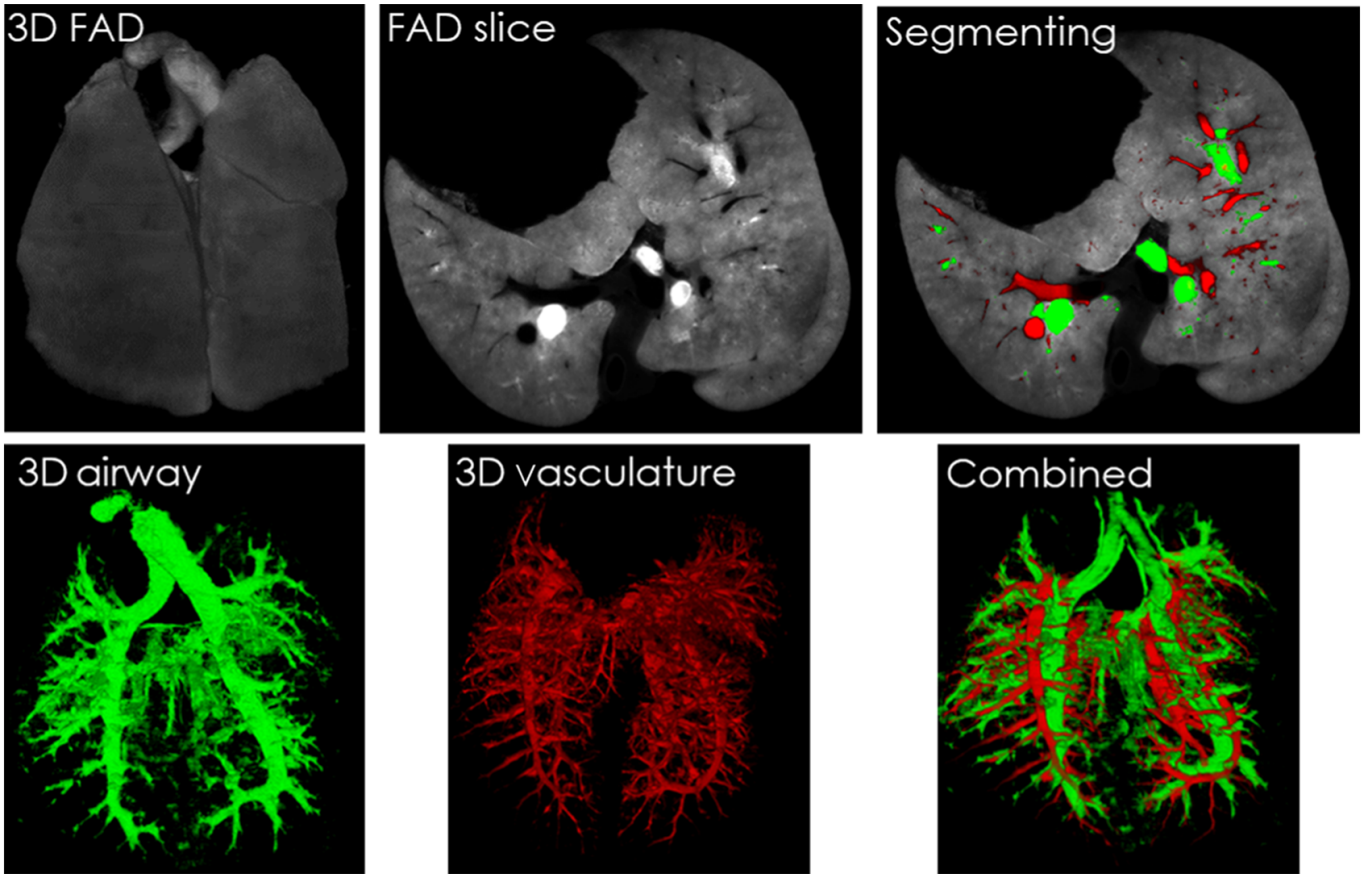
FITC airway injection helps to segment background vasculature from lungs. For a rat lung, FITC-dextran solution was injected into the airway. A raw 3D image of FAD fluorescence of lung is shown. A transverse slice view of raw lung image is chosen, and the segmented airway from light voxels of FAD image and vasculature from dark voxels are shown in green and red, respectively. Segmented airway and vasculature are merged with the raw slice to show the localization of the vascular and airway pixels in the segmenting image. Also the 3D vasculature and airway are combined and shown zero intersection (yellow voxel) with each other. Videos from 3D rendered images of FAD, airway, vasculature, and combined images can be found in Videos S5 (Video S5, 722 kB, MP4 [URL: https://doi.org/10.1117/1.JBO.26.7.076002.5]), Video S6 (Video S6, 753 kB, MP4 [URL: https://doi.org/10.1117/1.JBO.26.7.076002.6]), Video S7 (Video S7, 742 kB, MP4 [URL: https://doi.org/10.1117/1.JBO.26.7.076002.7]), and Video S8 (Video S8, 752 kB, MP4 [URL: https://doi.org/10.1117/1.JBO.26.7.076002.8]), respectively.

Vascular segmentation from the background of autofluorescence in lung tissues was not feasible because the vasculature, airway, and alveoli appeared dark in the images. Therefore, distinguishing the vasculature from these structures was not possible. To circumvent this problem, we injected an FITC solution into the airway and alveoli. Extrinsic fluorescence from FITC (excitation at 494 and emission at 537) and FAD (excitation at 437 and emission at 537) overlapped. This overlap and the injection of an FITC solution into the airway and alveoli enabled us to make the airway voxels bright in FAD images and keep the vascular structures dark. The same proposed segmentation algorithm was then applied to extract the inverted vasculature from the FAD images of the lungs. Figure 5 shows a 3D raw FAD image of the lung and a single slice of the lung. The airways, which are filled with FITC solution, are segmented from light (higher intensity) voxels in the FAD images. The 3D vasculature (in red) and airway (in green) are then reconstructed in 3D as shown in (Fig. 5). In the combined or merged images, the color of voxels that have an overlap between the segmented airway and vasculature should be yellow, but due to a very little intersection, no yellow voxels appeared in this figure. The Dice coefficient <0.001 also confirms that the airway and the vasculature did not overlap. These results demonstrate that the segmentation structures from inverted FAD images do not originate from airways but the vasculature. Note that now, the FAD images are originating from both FITC and FAD fluorescence. This helped us to lighten the airway, but the FITC fluorescence in the airway also interfered with the FAD signal. Therefore, on the downside, the RR is now NADH/(FAD + FITC), which is not an accurate representation of the mitochondrial RR (NADH/FAD).

### 3.2 Co-Registration with TdTomato to Confirm VMI Vasculature

**Fig. 6:**
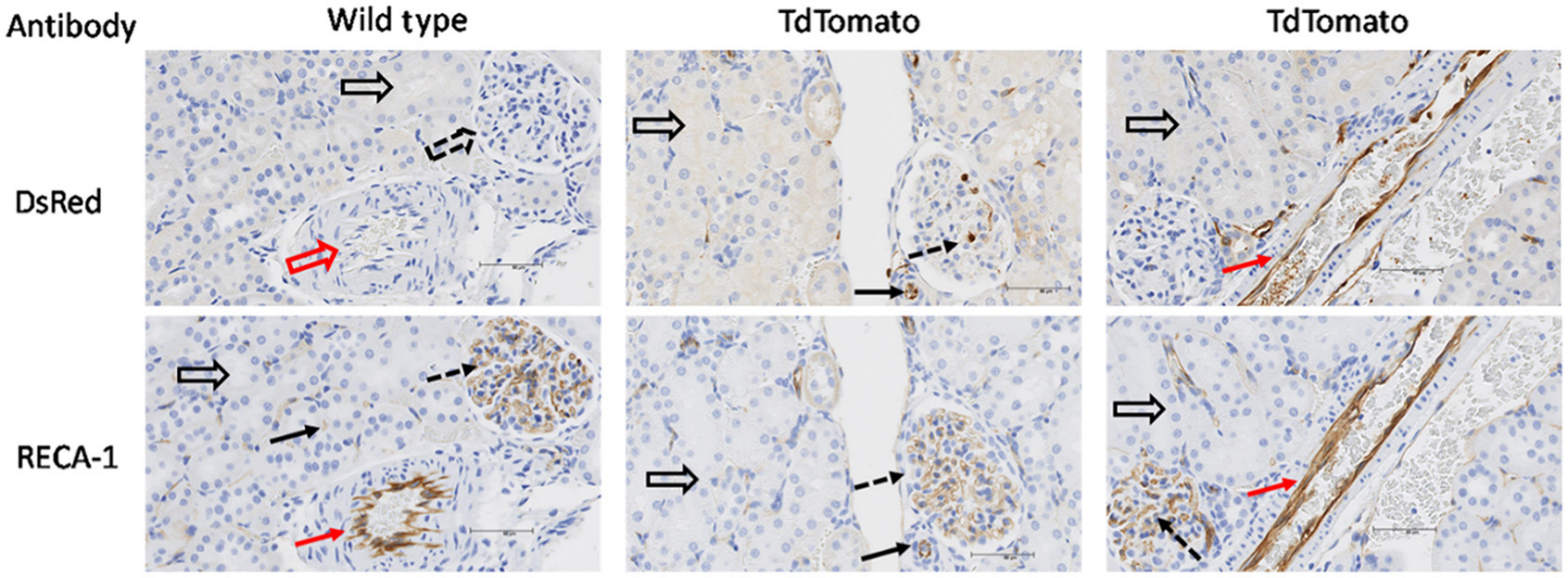
Vascular endothelial cells in transgenic but not wild type rats express TdTomato. This figure shows stained sections of kidneys from wild type and transgenic (TdTomato) rat. The upper panel represents sections stained with DsRed antibody specific for TdTomato (stained in brown). The lower panel represents adjacent sections stained for the endothelial marker RECA-1 (brown stain in lower panel). Note positive staining for TdTomato in the endothelial cells of the glomeruli (dotted arrow) as well as large (red arrow) and small (black arrows) blood vessels in the upper panel of transgenic rats. Wild type and TdTomato transgenic vessels in glomeruli (dotted arrows) and large (red arrow), and small (black arrows) vessels in the kidney also stain brown with endothelial-specific antibody RECA-1 (lower panel) confirming the brown staining in the TdTomato transgenics co-register with blood vessels. Open black arrows point to unstained tubules, open dashed arrows point to unstained glomeruli and open red arrows show unstained vasculature. Note the absence of TdTomato brown staining in glomeruli of wild type rats as well as tubules of TdTomato transgenic and wild type rats.

The transgenic rat model expressing endothelial-specific TdTomato was used to validate the vascular segmentation by VMI. Figure 6 shows a histological assessment that illustrates the expression of TdTomato in endothelial cells in transgenic rat kidneys (upper row). The renal tubules and most non-endothelial cells of the transgenic TdTomato rat kidneys do not express TdTomato (Fig. 6). Both wild type and transgenic vascular endothelial cells are also stained with endothelial-specific antibody RECA-1 in sections adjacent to those stained for TdTomato. Though the TdTomato-staining in glomeruli was not always as distinct as that of RECA-1, the sections demonstrated co-registration primarily with blood vessels and not with renal tubules (open black arrows).

**Fig. 7:**
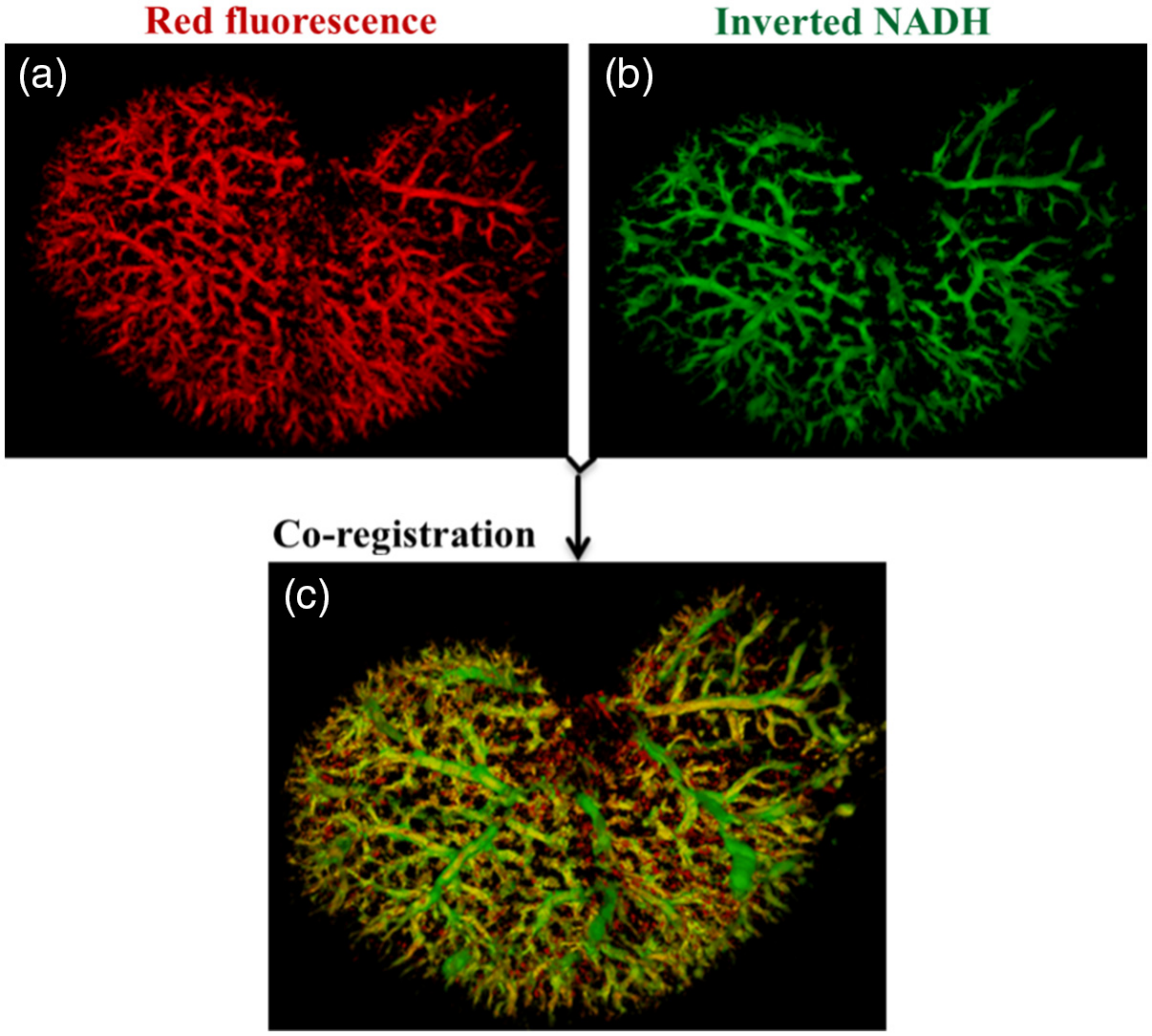
(a) TdTomato-transgenic rat kidney validates that VMI has high overlap with vessels expressing red fluorescence. The red channel is used as the ground truth for validating the vasculature extracted using VMI. (b) The segmented vasculature from light voxels of red channel and vasculature from the dark voxels of the NADH channel (VMI technique) are shown in red and green, respectively. (c) Segmented vasculatures are then combined to localize their intersection in yellow. The dice coefficient of 0.91 shows a great precision in vascular segmentation.

Using the TdTomato transgenic rat model, the cryo-imaging was performed in the two channels of fluorescence, NADH (excitation 350 nm and emission at 460 nm), and red (excitation 545 and emission 645). The bright voxels in the red channel (segmentation algorithm without step 2) and the dark voxels in the NADH channel are segmented and reconstructed [Figs. 7(a) and 7(b), respectively]. In the kidney, the anatomy of the vasculature extracted from the NADH using VMI [Fig. 7(a)] is then combined with the vasculature segmented from red fluorescence [Fig. 7(b)] to make a hybrid image [Fig. 7(c)]. The overlap voxels between the two images [Figs. 7(a) and 7(b)] are displayed in yellow color [Fig. 7(c)]. The co-registration gives a Dice coefficient of 0.91, which shows a high degree of overlap/merge between the two segmented vasculatures.

**Fig. 8:**
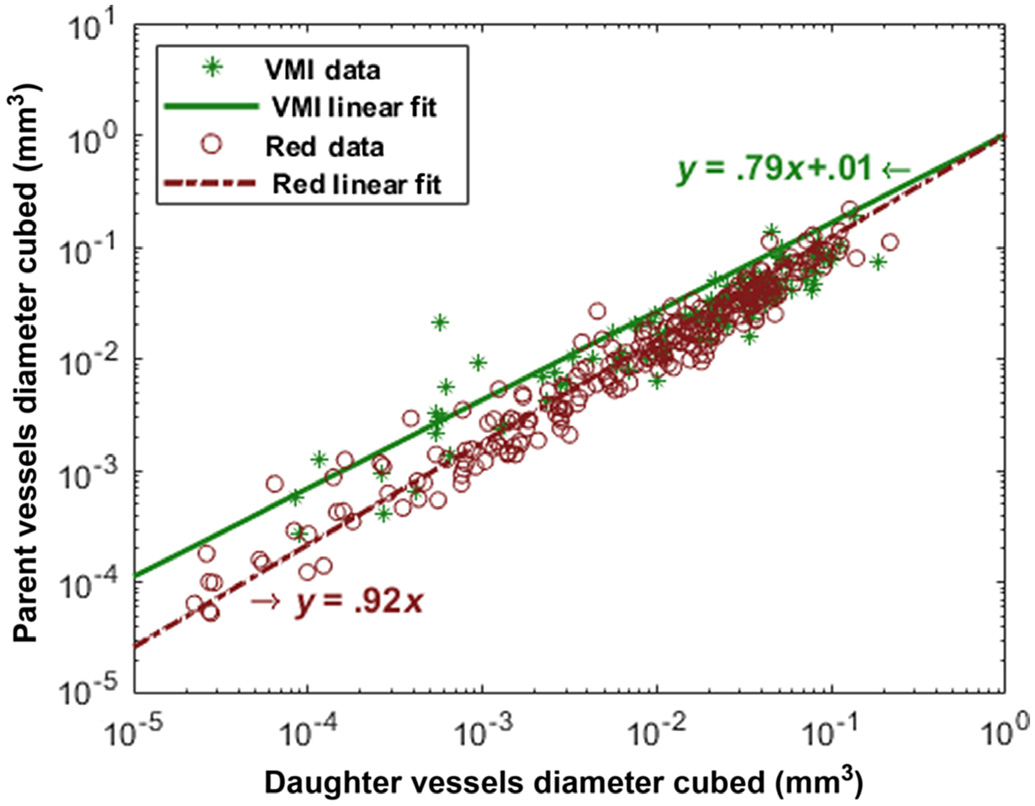
VMI follows Murray’s Law. The parent vessel diameter cubed is plotted against the sum of the diameter cubed of their corresponding daughter vessels. The data from red fluorescence of TdTomato-transgenic rat kidney are shown as red circles, and the data from vasculature extracted using the VMI technique are represented as green stars. The two vascular branching data from VMI and red fluorescence have merged, and their linear regression fit is compliant with Murray’s law (identity line, y=x).

The branching of the vasculature between the two signals is also compared in Fig. 8. The relationship between the cubed diameter of the parent vessels to the summation of the cubed diameter of their corresponding branched daughter vessels is presented. Using linear regression, the two lines are fitted to each set of data points as shown in Fig. 8. According to Murray’s law, the data should be fitted to y=x line, i.e., a line with a slope of 1 and y intercept of 0. The y intercept for both lines is ∼0, and the slopes for both VMI and red channel are close to 1, indicating that the VMI branching like the red vascular branching follows Murray’s law of the “minimum work” hypothesis successfully. A single branch from the two signals is also evaluated for more insights into smaller vessel branches, and both VMI and red vascular branching follows Murray’s law on smaller branches as well (the data are provided in Fig. S2 in the Supplementary Material).

### 3.3 VMI of Organs from Partial Body Irradiated Rats

**Fig. 9:**
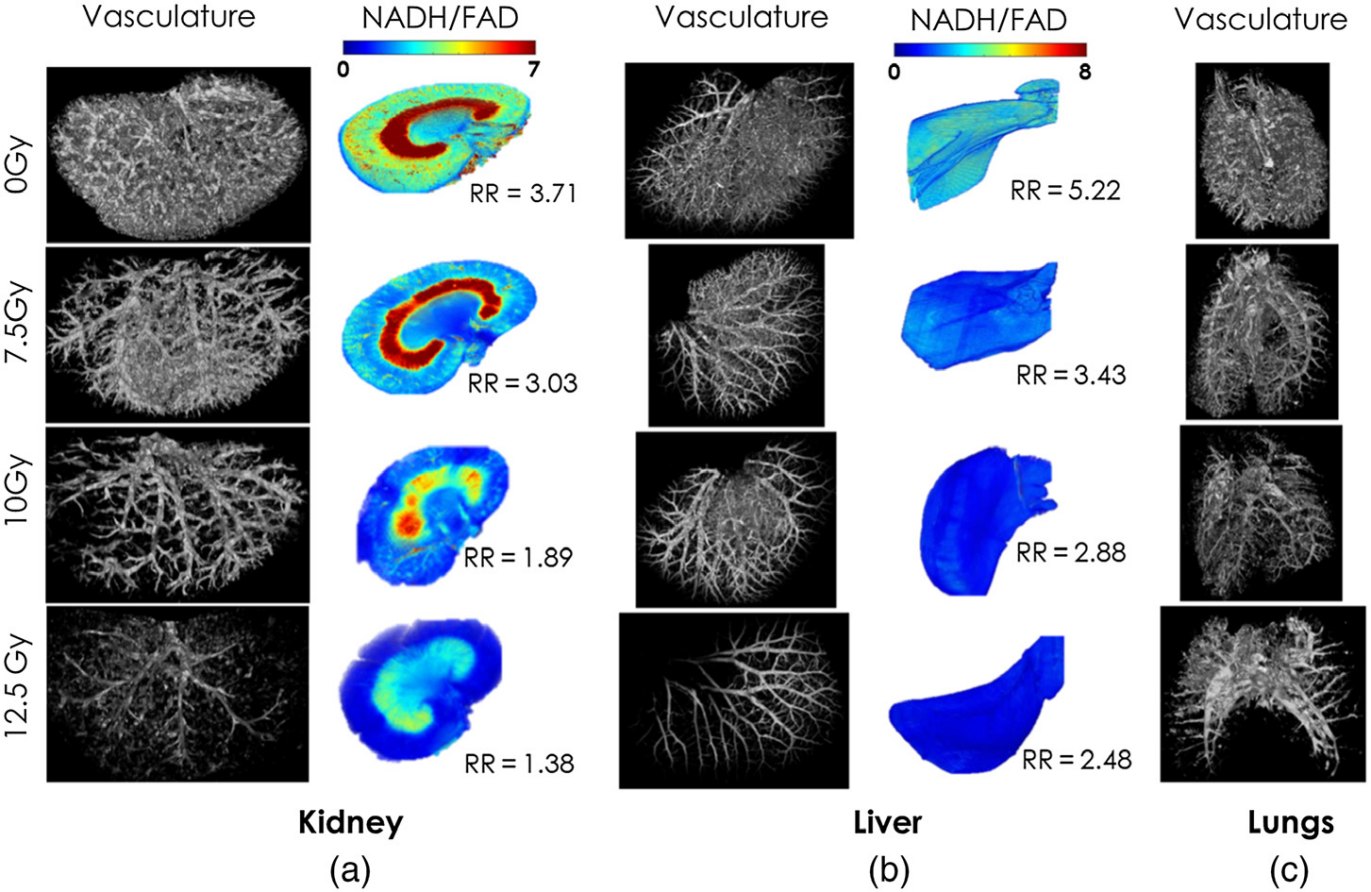
Increasing doses of PBI resulted in increased severity of vascular-metabolic damage in (a) kidneys, (b) livers, and (c) lungs. The dose of irradiation decreases the RR (NADH/FAD). The RR images are presented in pseudo-color with higher RR voxels shown in red and lower RR voxels in blue. The mean RR is also provided by the images.

Here we present an application of VMI to uniquely drive the topography of two sets of parameters simultaneously: mitochondrial redox state and the 3D vascular network of whole organs. Figure 9 illustrates the representatives of the 3D rendered vascular networks of the kidney, liver, and lung from rats exposed to different doses of irradiation. The corresponding 3D RR (NADH/FAD) of the kidney and liver are also presented in Fig. 9. The RR images of the lungs are not presented due to the interference of FITC with FAD.

The vascular networks in Fig. 9 illustrate the regression of the vessel networks after PBI. The vascular damage in kidneys and lungs also appears to qualitatively correlate with the dose of irradiation in the PBI rats. The RR images are presented in pseudocolor with higher RR voxels shown in red and the lower RR voxels in blue. The kidneys and livers exposed to a higher dose of irradiation show a greater decrease in the RR, representing a more oxidized mitochondrial redox state.

## 4 Discussion

Due to the low levels of autoflourescence signals in tissue autofluorescence metabolic imaging, the autofluorescence images have limited tissue contrast anatomically when compared to the histology images. This limitation was partially circumvented in the current study using VMI to provide a 3D vascular network of a whole organ. Here we demonstrate the feasibility of VMI to generate anatomical and metabolic information simultaneously. The dark voxels inside the autofluorescence images were segmented to provide the 3D vascular network of whole organs. The injection of exogenous fluorescent dye into the airway of the lungs helped to highlight the airway so that the dark voxels solely represent the vasculature.

The organ-level vasculature is the focus and strength of our study, which is hard to achieve with existing technologies. There are multiple vascular imaging modalities such as OCT that can be used on small field of view, while VMI can provide the whole-organ vascular structure. Like VMI, Kaushik et al.^47^ performed vascular imaging using autofluorescence signal. However, Kaushik et al. performed imaging on engineered tissue using synthetic hydrogel, while VMI imaged frozen rodent organs with blood and tissue around vascular structures. This would explain the difference in the two methods: Kaushik et al. perform vascular imaging using NADH signal from synthetic vasculature, while VMI uses light-absorbing properties of hemoglobin and invert the NADH image to segment the vasculature.

It was also shown that the VMI has high co-localization with the red fluorescence of transgenic rats expressing endothelial-TdTomato. A genetically modified rat model of vascular endothelium selective expression has been chosen to confirm the selection of the vasculature by VMI. The high overlap/merge between the red fluorescence of transgenic-TdTomato rat kidney and VMI vasculature indicates the specificity of VMI in the segmentation of vascular networks. Also, we have shown that the “minimum work” hypothesis proposed by Murray^46^ has been satisfied by both approaches. This suggests that the VMI vasculature has similarities in branching with the ground truth vasculature that was generated by TdTomato red fluorescence.

The potential interest of combining exquisitely sensitive autoflourescence metabolic information with vascular information was demonstrated in a proof-of-concept study of radiation-induced damage to multiple organs. 3D mitochondrial redox state of PBI rat kidneys and livers were examined. The mitochondrial redox state of kidneys and livers appear to decrease in an irradiation dose-dependent manner. This result is consistent with our previous study^40^ showing that irradiation diminished the ability of the cells to maintain balanced mitochondrial redox state necessary for normal bioenergetics in kidneys. Using VMI, the vascularization during exposure to different doses of irradiation was examined in the kidneys, livers, and lungs. We have seen that exposure to irradiation could also cause vascular regression. Comparing the observed radiation-induced vascular damage with the previously seen impact of radiation on potentially increased oxidation of the mitochondrial RR^40^ implies a link between the deregulation of mitochondrial metabolism and the regression of the vasculature typical of radiation injuries.^31^^–^^33^ Together, this study showed that VMI using autofluorescence can successfully stratify the dose of irradiation based on these two biomarkers of injury.

The vascular segmentation algorithm in VMI uses the same 3D autofluorescence cryo-images that we have used previously to produce tissue mitochondrial redox state.^26^^,^^40^^,^^48^^,^^49^ VMI can be applied to quantitatively characterize the organ vasculatures and the metabolic state simultaneously. The VMI can also be used to explore the pathophysiology of rodent injury and treatment models. Optical metabolic imaging has been applied for several years,^26^^,^^40^^,^^48^^–^^52^ and by adding the proposed segmentation technique, another key biomarker of injury, vascular density, would also be measured.

The major limitation of this study is that VMI has only been, to date, applied to autofluorescence images of frozen tissue. The application of the technique on *in vivo* autofluorescence images has not been studied. In FITC airway injected lungs, due to the interference of FITC with the FAD signal, accuracy of the mitochondrial redox imaging in the lung may be compromised. A challenge in performing VMI on hearts was that there are cavities that needed to be masked, the optional step 5 has been added to segment out the unwanted spaces (Fig. 1).

The proposed algorithm in this study has generated both vascular and metabolic information with major implications.

(a) The vascular images are produced without the use of any extrinsic contrast agents or tissue clearing-solvents, which might induce artifacts and/or structural deformity.^53^
(b) In multimodal imaging technologies, the co-registration of metabolic and vascular images is of paramount importance.^54^ VMI has perfect co-registration precision because vascular and metabolic images are originating from the same 3D images, making it ideal for studying the interaction of tissue metabolism and vasculature.
(c) In this study, vascular images of the kidney, lung, heart, and liver were segmented using VMI. This VMI approach could be extended to other organs and pathologies, such as eye, skin wounds, and tumor as we^48^^,^^50^^,^^55^ and others^56^ have investigated their mitochondrial RR in the previous reports.
(d) Since the technique uses optical imaging technologies, it is capable of high-resolution imaging compared to x-ray or ultrasound instruments. By increasing the resolution of the fluorescence cryo-imaging instrument, VMI can present additional details in the vascular networks. Also unlike the laborious, complex, and time-consuming sample preparations in micro-CT,^15^ the only sample preparation in VMI before performing fluorescence imaging is snap-freezing the tissue in liquid nitrogen.
(e) VMI is implemented by adding an image processing algorithm to the existing 3D fluorescence cryo-imaging. Therefore, no hardware modification is needed to extract the vascular network of organs from autofluorescence. Also fluorescence metabolic imaging systems are much more cost-effective in comparison to other similar 3D whole organ vascular imaging modalities, such as micro-CT.
