# Supplementary material for: Three-dimensional vascular and metabolic imaging using inverted autofluorescence
Source: J Biomed Opt. 2021 Jul 8;26(7):076002. doi: 10.1117/1.JBO.26.7.076002 (PMC8265174; doi:10.1117/1.JBO.26.7.076002)
Supplement: Supplementary file 1 [file JBO_026_076002_SD001.pdf]

Table S1: A simple implementation steps of the segmentation algorithm using Fiji.

Steps	Implementation
**Input**	*File* $\rightarrow$ *Import* $\rightarrow$ *Image Sequence*
**Step 1**	*Image* $\rightarrow$ *Adjust* $\rightarrow$ *Brightness/Contrast* (adjust the minimum and maximum)
**Step 2**	*Edit* $\rightarrow$ *Invert*
**Step 3**	*Process* $\rightarrow$ *Subtract Background* (set the radius based on the largest vessel)
**Step 4**	Repeat step 1
**Step 5**	(optional) Mask out unwanted regions, the mask can be found by thresholding
**Step 6**	(For quantification) Use Filament tracing module in Imaris or any conventional 3D vessel tracing algorithm available

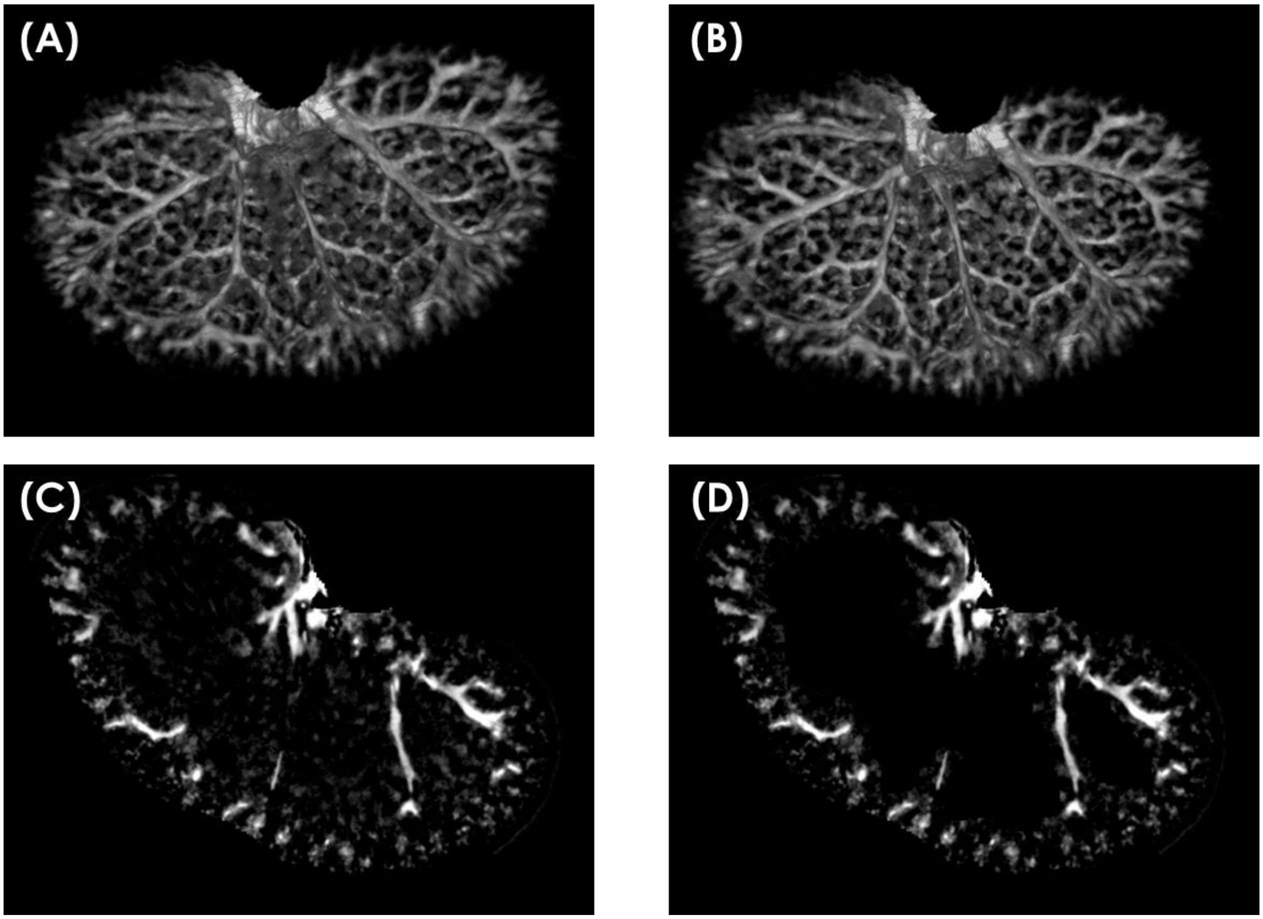

Fig. S1 3D vascular image of a half kidney before (A) and after (B) medullary-region removal. The slice view is also shown before (C) and after (D) removal for comparison.

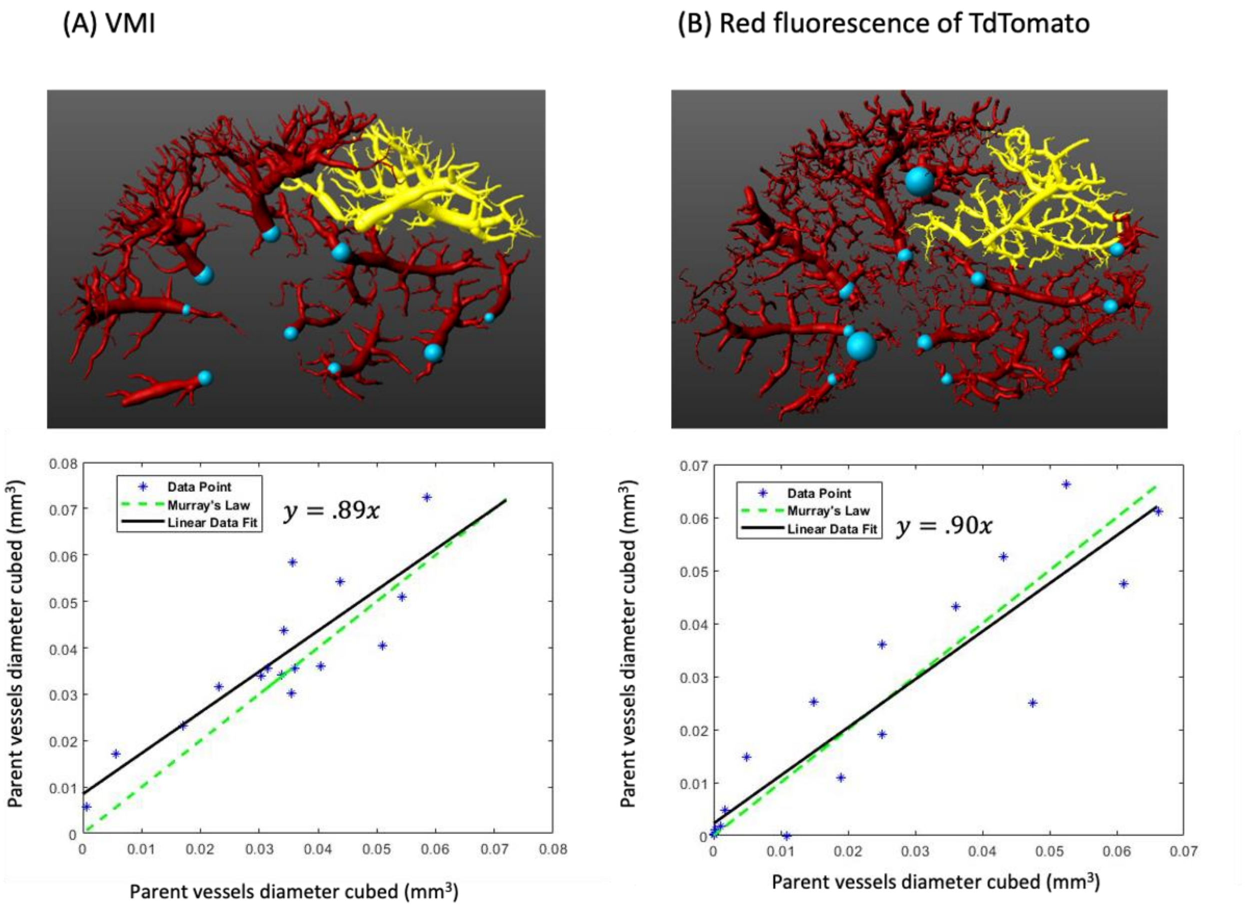

Fig. S2 Extracted vascular images in Imaris using (A) VMI and (B) red fluorescence of TdTomato rat kidney are shown in top row. A single branch is chosen (in yellow) to perform single branch structural evaluation. The bottom row is the comparison of parent-daughter vessel diameter relationship in the segmented vasculatures.
